# Surfactant Protein A in Cystic Fibrosis: Supratrimeric Structure and Pulmonary Outcome

**DOI:** 10.1371/journal.pone.0051050

**Published:** 2012-12-07

**Authors:** Matthias Griese, Stephanie Heinrich, Felix Ratjen, Michael Kabesch, Karl Paul, Manfred Ballmann, Ernst Rietschel, Matthias Kappler

**Affiliations:** 1 Dr. von Haunersches Kinderspital, University of Munich, Munich, Germany; 2 Paediatric Respiratory Medicine, Hospital for Sick Children, Toronto, Ontario, Canada; 3 Department of Pediatric Pneumology, Allergy and Neonatology, Hannover Medical School, Hannover, Germany; 4 Lungenpraxis, Berlin, Germany; 5 St. Josefs Hospital, Klinik für Kinder- und Jugendmedizin, Bochum, Germany; 6 Klinik und Poliklinik für Kinder- und Jugendmedizin, University of Cologne, Köln, Germany; University of Tübingen, Germany

## Abstract

**Background:**

The state of oligomerization of surfactant associated protein-A (SP-A) monomers differs between individuals. This likely affects SP-A’s functional properties and could thereby influence clinical status in patients with lung diseases. In this study we focus on SP-A structure in cystic fibrosis (CF) compared to both healthy subjects and disease controls.

**Methods:**

SP-A composition and function were assessed in both bronchoalveolar lavage (BAL) fluid and serum of 46 CF patients with mild disease, 25 patients with chronic bronchitis and 22 healthy subjects by gel chromatography and a functional agglutination assay. Relation of SP-A agglutination ability to disease severity of the subjects was explored.

**Results:**

SP-A was present in seven major oligomeric forms with the majority of SP-A being structurally organized as complex oligomeric forms. More complex oligomeric forms were associated with better SP-A function with regard to its agglutination ability. These forms were more frequently observed in BAL than in serum, but there were no differences between disease groups. In CF patients, more complex forms of SP-A were associated with better lung function.

**Conclusions:**

Organizational structure of SP-A affects its functional activity and is linked to disease severity in CF.

## Introduction

Surfactant associated protein-A (SP-A) is the most abundant pulmonary surfactant protein and belongs to the family of innate host defense proteins termed collectins. SP-A is involved in surfactant metabolism and recognizes pathogen-associated molecular patterns on microorganisms, resulting in aggregation, opsonization or permeabilization of microorganisms and facilitation of microbial clearance [1]. Intermolecular disulfide bonds at the N-terminal end enable the aggregation of trimers to higher oligomers, whereas the neck region arranges the trimer association of the globular domain and influences the binding to phospholipids and bacterial lipid A. Functional properties of SP-A may depend on its supratrimeric assembly and organization [2]–[4]. While the relevance of the higher-order oligomerization of lung collectins for their functional capabilities has been extensively studied for SP-D [5], little data is available for SP-A multimers [4], [6], [7].

Cystic fibrosis (CF) is a life limiting disease associated with chronic pulmonary infections [8]. Bronchoalveolar lavage (BAL) levels of SP-A have been shown to be increased early in the course of the disease [9], but decrease as disease progresses and lower levels are correlated with more inflammation and diminished lung function [10]–[15]. Currently, there are no data available on macromolecular organization of SP-A in BAL and its potential functional role in CF airway disease.

The aims for this study were to assess structural organization in CF compared to other airway diseases and controls, relate organization to functional activity in vitro, and to explore associations between SP-A organization and function with clinical markers of disease severity in CF patients.

## Methods

### Ethics Statement

The study was approved by the institutional review board, the Ethics committee of the Medical Faculty of the LMU Munich (EK 026-06) and all participants gave their written informed consent.

### Subjects

46 patients with CF, 25 patients with chronic bronchitis and 22 healthy control subjects (controls) were included in this study. Samples were collected in previous studies [13], [16], [17] and details of the patients are given in Table 1. We preferentially studied these only mildly affected patients in order to avoid secondary alterations caused by severe neutrophilic airway inflammation. Pulmonary disease severity was assessed according to Schluchter et al 2006 who used the mean change of FEV_1_ per year (ΔFEV_1_)(% pred.), allocation to lung disease severity group (mild/moderate/severe; i.e. mild, if FEV1 % pred. at the age of 20 years ≥ 84, severe, if ≤54) and the FEV1 at the age of twenty (FEV1 (% pred.)_age 20_), which was calculated from all FEV_1_ (% pred.) available and covered an average of at least 3 years before the latest sampling point with four values each year per patient [18].

**Table 1:** Clinical data of the subjects investigated.

Subjects’ characteristics	Cystic fibrosis	Chronic bronchitis	Controls
Age (years)	13±6 (n = 46)	11±12 ( n = 25)	16±9 ( n = 22)
Gender in % female (absolute numbers))	57 (26)	52 (13)	36 (8)
Body mass index in % overweight/normal/underweight (absolute numbers)§	20/47/33 (7/22/15)	17/83/0 (2/10/0)	0/100/0 (0/19/0)
FEV1 (% pred.)§§	94±26 (n = 46)	94±22 (n = 8)	118 (n = 1)
Mean change of FEV1 per year (ΔFEV1)(% pred.)§§§	–3±10 (n = 42)	n.k.	n.k.
Lung disease group in % mild/moderate/severe (absolute numbers)	72/0/28 (28/0/11)	91/9/0 (10/1/0)	100/0/0 (22/0/0)
P.aer. infection of the lung at sampling point (%)	24 (n = 11)	n.k.	n.k.
IgG in serum (ratio to upper normal value)	0.9±1.8 (n = 28)	0.5±0.4 (n = 10)	n.k.
Neutrophils in BAL (%)	28±22 (n = 39)**	7±6 (n = 11)	2±2 (7)
SP-A level in BAL (ng/ml)§§§§	5023±3314 (n = 39)	7698±6323 (n = 13)	6204±5111 (n = 8)
SP-A level in serum (ng/ml)§§§§	26±9 (n = 37)	36±14 (n = 6)	26±11 (n = 15)
Agglutinate size in BAL (Pixel) (18mers/6mers/trimers)	316±30/234±15/74±15 (n = 10)	370±43/316±19/114±13 (n = 10)	517±34/352±12/118±7 (n = 10)
Agglutinate size in serum (Pixel) (18mers/6mers/trimers)	481±35/380±32/146±13 (n = 10)	469±36/377±18/143±8 (n = 10)	525±31/361±15/132±7 (n = 10)

Data are mean ± SEM of n subjects or the percentage of subjects in a group (in brackets absolute number of subjects). Statistical comparisons were made by One-way ANOVA with post hoc test, Tukey ** indicates a significant difference (p<0.01) compared to chronic bronchitis and controls.

n.k.: not known.

§ BMI = body mass index (weight (kg)/(length (m2)). Age-dependent cut-off values used to define weight status groups (underweight, normal, overweight) were taken from the global database on body mass index (http://www.who.int/bmi/index.html).

§§ indicates FEV1 (% pred.)age 20 for patients with cystic fibrosis, calculated according to Schluchter et al 2006,

§§§ data were calculated from all mean FEV1 (% pred.) available and covered an average of at least 3 years before the latest sampling point with four values each year per patient.

§§§§ Serum and lavage samples were not available as pairs in all subjects; thus the numbers of each measurement are different.

There was no correlation between the neutrophils in BAL (%) or the IgG in serum (ratio to upper normal value) and the FEV1 (% pred.).

### BAL

BAL was performed with 4×1 ml/kg body weight normal saline warmed to body temperature [13]. The first aliquot of the recovered BAL fluid was treated separately; all other samples were pooled and used for analysis in this study.

### Gel Chromatography

**Figure 1:**
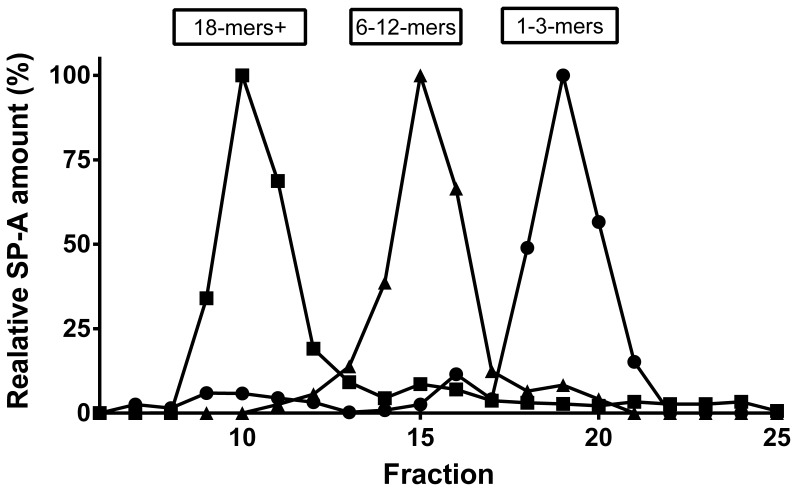
Illustration of the column characteristics for the separation of SP-A present in bronchoalveolar lavage or serum samples. Samples from different patients were run on the column and SP-A content of each fraction was determined and plotted, normalized to its total amount eluting from the column. Here, selected samples from three different subjects, each with a distinct extreme pattern of elution, are shown (patient A (squares), having mainly one initial peak around fraction 10 contains 18-mers and larger; patient B (triangles), having mainly a peak around fraction 15 with SP-A 6–12-mers; and patient C (circles), having mainly the lower molecular weight forms of SP-A).

To determine the structural organization of SP-A in BAL and serum gel chromatography on a superpose 6 column was used as described in previous studies [6], [7]. This method separates SP-A molecules with regard to their oligomerization form. The column was calibrated by using blue dextran (2000 kDa), holotransferrin (669 kDa) and bovine serum albumin (66 kDa)(not shown). Samples of 1 ml of BAL fluid and 500 µl of serum were loaded. The SP-A content of the eluted fractions (800 µl) was determined by Slot-Blot assay. The void volume of the column eluting between fractions 8 and 9 was about 6.8 ml. SP-A usually elutes as three distinct peaks, ranging from fractions 9–12, 13–17 and 18–22 (Fig. 1). The first peak contains the largest SP-A multimers, i.e. 18 mers and bigger, the second peak 6 to 12 mers and the third peak di- and trimers. A major peak was defined to be present if the SP-A amount was at least or more than 20 % of the total SP-A eluted from the column and each peak was separated from the previous by a distance of 5 fractions of 0.8 ml each. The cut off of 20% was arbitrarily selected after visual inspection of all chromatograms normalized into a grid. There were baseline fluctuations and drifts up to 10% and the level of 20% of total SP-A was selected to define the presence or absence of a peak in the preset elution fractions. There were a total of 140 chromatograms which had a potential for 177 changes in pattern (i.e. 010 can improve or deteriorate, etc). Lowering the threshold to 10%, would introduce 15 changes in the category of the pattern in both directions and a net change of 5 improvements. The threshold set also makes sure that a peak is represented by a sufficient amount of the total SP-A.

Depending on the presence (1) or absence (0) of any of the three major peaks in a sample (Fig. 1), generally 7 different patterns of SP-A can be classified (“Code” in first column of Table 2). The inter-run reproducibility of the separation of SP-A by the gel chromatography and determination of SP-A concentration by slot-blot was assessed in four serum and 2 BAL samples of 5 different individuals by repeated analysis at intervals of four weeks. The reproducibility of the gel chromatography was excellent comparing the curve forms, the coefficient of variation for SP-A content assessed by the area under the curve of the peaks was 29%.

**Table 2:** Oligomeric distribution of SP-A in BAL and serum.

Col #1	#2	#3	#4	#5	#6	#7	#8	#9	#10	#11	#12
	BAL					Serum					
Code	Cystic fibrosis	Bronchitis	Controls	Differences pt-groups (col #2, 3, 4)	All Pts (mean)	Cystic fibrosis	Bronchitis	Controls	Differencespt- groups(col #7, 8, 9)	All Pts(mean)	DifferencesBAL - serum(col #6, 11)
Peaknumber1–2–3Present = 1Absent = 0	n = 42	n = 21	n = 18	p-value	(% present)	n = 34	n = 14	n = 11	p-value	(% present)	
0–0–1	2 (5 %)	3 (14 %)	0 (0 %)	NS	6.3	0 (0 %)	3 (21 %)*	0 (0 %)	0.0062	7.0	NS
0–1 - 0	0 (0 %)	3 (14 %)	2 (11 %)	NS	8.3	7 (21 %)	0 (0 %)	7 (64 %)	NS	28.3	0.0049
0–1–1	1 (3 %)	0 (0 %)	1 (6 %)	NS	3.0	3 (9 %)**	4 (30 %)	4 (36 %)	<0.0001	25.0	0.0019 (0.004)
1–1–1	11 (26 %)	1 (5 %)	5 (28 %)	NS	19.7	5 (15 %)	2 (14 %)	0 (0 %)	NS	9.7	NS
1–1 - 0	13 (31 %)	6 (29 %)	1 (6 %)	NS	22.0	18 (52 %)**	3 (21 %)	0 (0 %)	0.0028	24.3	NS
1 – 0–1	7 (17 %)	4 (19 %)	7 (39 %)	NS	25.0	0 (0 %)	2 (14 %)	0 (0 %)	NS	4.7	0.0013 (0.003)
1 - 0 - 0	8 (18 %)	4 (19 %)	2 (10 %)	NS.	15.7	1 (3 %)	0 (0 %)	0 (0 %)	NS.	1.0	NS
Sum =	42 (100 %)	21 (100 %)	18 (100%)			34 (100%)	14 (100%)	11 (100%)		100.0	
Peak 1present in	39 (93 %)	15 (71 %)	15 (83 %)	NS	85.2	24 (71 %)**	7 (50 %)	0	0.0002	52.5	<0.0001 (<0.0001)
Peak 2present in	25 (60 %)	10 (48 %)	9 (51 %)	NS	54.3	33 (97 %)	9 (64 %)	11 (100%)	NS	89.8	<0.0001 (<0.0001)
Peak 3present in	21 (50 %)	8 (38 %)	13 (73 %)	NS	51.8	8 (23 %)	11 (79 %)**	4 (36 %)	0.0018	38.9	NS

Samples were separated by gel chromatography and the fractions were analyzed for SP-A content by Slot-Blot. Given is the absolute number and in brackets the percentage of patients with a particular distribution pattern.

Differences between the frequency of the oligomeric pattern distribution of SP-A are given in columns (Col) #5 for BAL and col #10 for serum and were calculated by Chi-square-test for 3 groups, the p-values are indicated, if <0.05.

SP-A present in each of the fractions from gel chromatography was determined by slot blot (see below) and the amounts of the different structures were calculated from the area under the curve of the corresponding peak as a percentage of total amounts of SP-A present (Fig. 1).

### Slot Blot and SP-A Self-agglutination Measurement

The SP-A concentration of chromatography fractions was determined by the slot-blot method. An assay previously designed to assess the ability of SP-A in serum and BAL samples to agglutinate, was used [19]. Briefly, anti-SP-A antibodies (polyclonal recombinant rabbit-anti-human SP-A antibody (Nycomed, Konstanz, Germany) which bind SP-A at its N-terminal end were coupled to 0.05 µm latex beads, so that the bound SP-A was able to interact by its C-terminal CRD with other SP-A molecules in a given sample. This causes agglutination of the beads forming larger aggregates, which were observed by light microscopy. The agglutinates were photographed at 10 fold magnification, the pictures viewed with Adobe Photoshop software and the size of all the agglutinates (typically more than 100) was assessed and the largest measured in pixels by drawing squares around them and averaged by a blinded examiner with regard to the diagnosis [19].

## Results

Clinical characteristics of the study population are shown in Table 1. CF patients included in this study had mild lung disease as indicated by a normal mean FEV1 and the majority had a normal nutritional status expressed as BMI. The rate of P. aeruginosa positive cultures was low and mean serum IgG, a marker of the systemic inflammatory response, was within the normal range. In contrast to the controls and the subjects with chronic bronchitis, the CF patients had moderately increased neutrophilic counts (Table 1). The total levels of SP-A in serum or BAL were not different between the patients with CF and those with bronchitis and the controls (Table 1).

In BAL there were no differences in the oligomeric distribution of SP-A between the 3 study populations (Table 2). Complex oligomeric forms of SP-A such as octadecamers (first peak) were the most prevalent form (85% of all subjects), followed by smaller oligomeric forms such as hexamers (54% of all subjects, second peak), and dimers or trimers (52% of all subjects, third peak).

In serum, all controls showed dimers and trimers, smaller and larger oligomeric forms were observed at a lower rate. Interestingly, octadecamers (present above the threshold defined at 20% of the SP-A in a sample) were infrequently identified in sera of control patients (Tables 1 and 2).

No correlation in oligomer pattern between corresponding serum and bronchoalveolar lavage samples was observed, regardless of whether the overall pattern (Tab. 2) or individual peaks were considered (data not shown).

**Figure 2:**
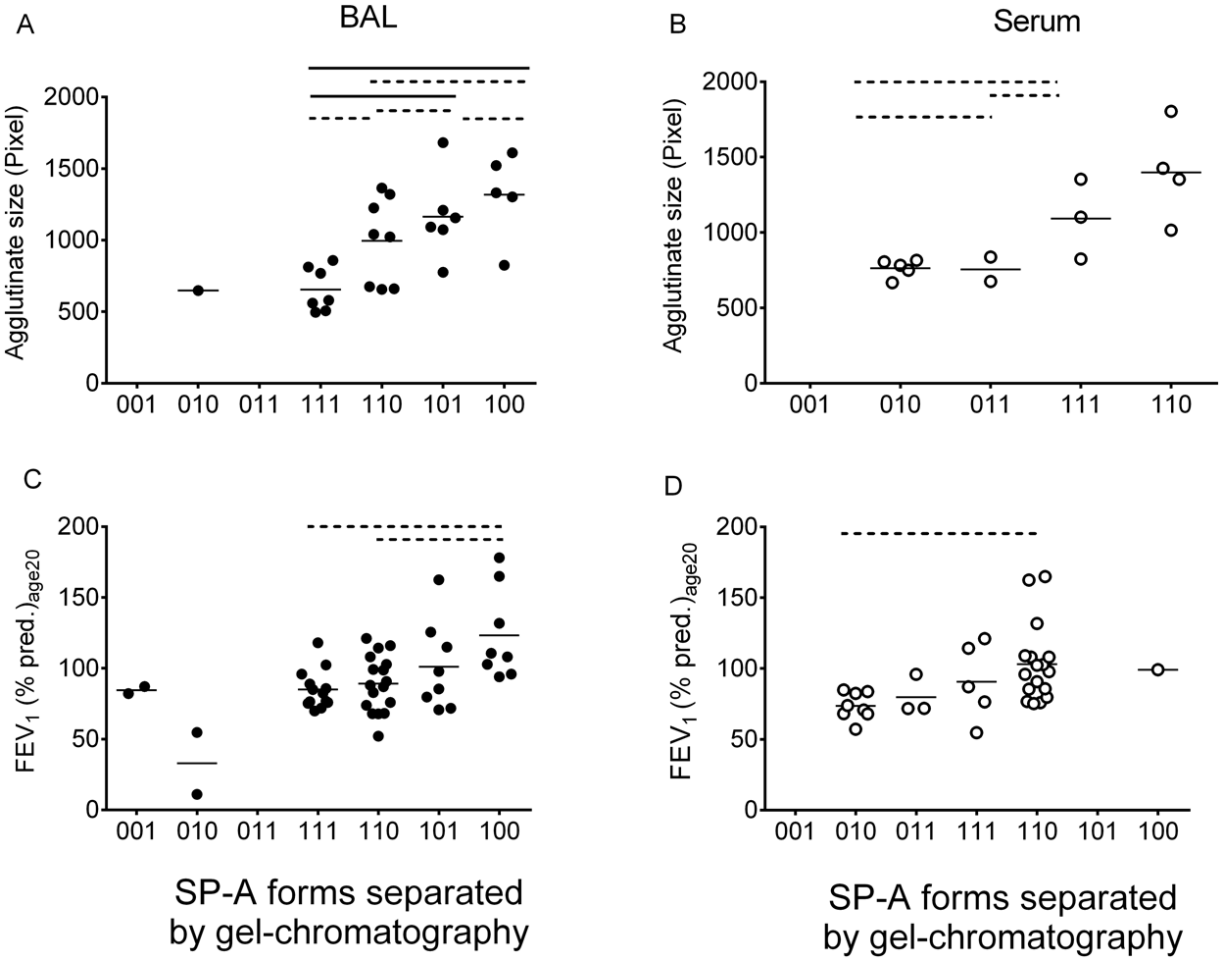
Relationship between different structure compositions of SP-A and their agglutination ability (A,B) as well as their association with lung function (C,D). The graphs illustrate the agglutination ability differences between different oligomeric structure compositions (forms) in BAL (A) and serum (B) analyzed by gel chromatography. 42 BAL samples and 31 serum samples of CF patients were analyzed. In serum only the 010 and 110 forms showed a significant difference regarding the agglutination ability compared to other forms. In BAL the 100 form agglutinated significantly better than the 111, 110 and 101 form and the 101 form significantly better than the 111 and 101 form. The graphs (C,D) plot the relation between the lung function (y-axis) and the oligomeric SP-A distribution in BAL (C) and in serum (D). For analysis One way ANOVA with Tukey as post-test was used and in all cases with a significant result (P<0.05), post hoc tests were calculated. Differences between groups are indicated by dotted (P<0.05) or solid (P<0.01) lines.

As expected in CF patients neutrophils and elastase activity were elevated in CF BAL fluid (36±38 U/ml, n = 18, compared to normal reference where there is no free elastolytic activity [20], [21]. However, not all patients had activity in their BAL and there was only a weak (P<0.05, r = 0.478) correlation between elastase and SP-A present as dimers/trimers (%), but not to the higher oligomeric forms or the pattern frequencies. No correlations were found with the neutrophils (%).To assess the relationship between oligomeric organization of SP-A and its function in CF patients, a functional rank order regarding organizational structure ranging from more to less complex structure was generated (001<010<011<111<110<101<100). As shown in Fig. 2 a+b more of the complex forms were associated with larger agglutinates. To analyze whether these correlations were caused by structural composition of the whole samples, or the composition of an individual fraction of a sample, individual fractions were isolated from patients’ samples and analyzed at equal SP-A concentrations for their ability to agglutinate. No differences in agglutination activity were observed for the same oligomeric forms, isolated from subjects with different oligomeric pattern (data not shown). This suggested that rather than potential differences among the oligomeric forms within a particular fraction, the relative composition of different oligomeric forms in a given sample is responsible for its activity.

**Figure 3:**
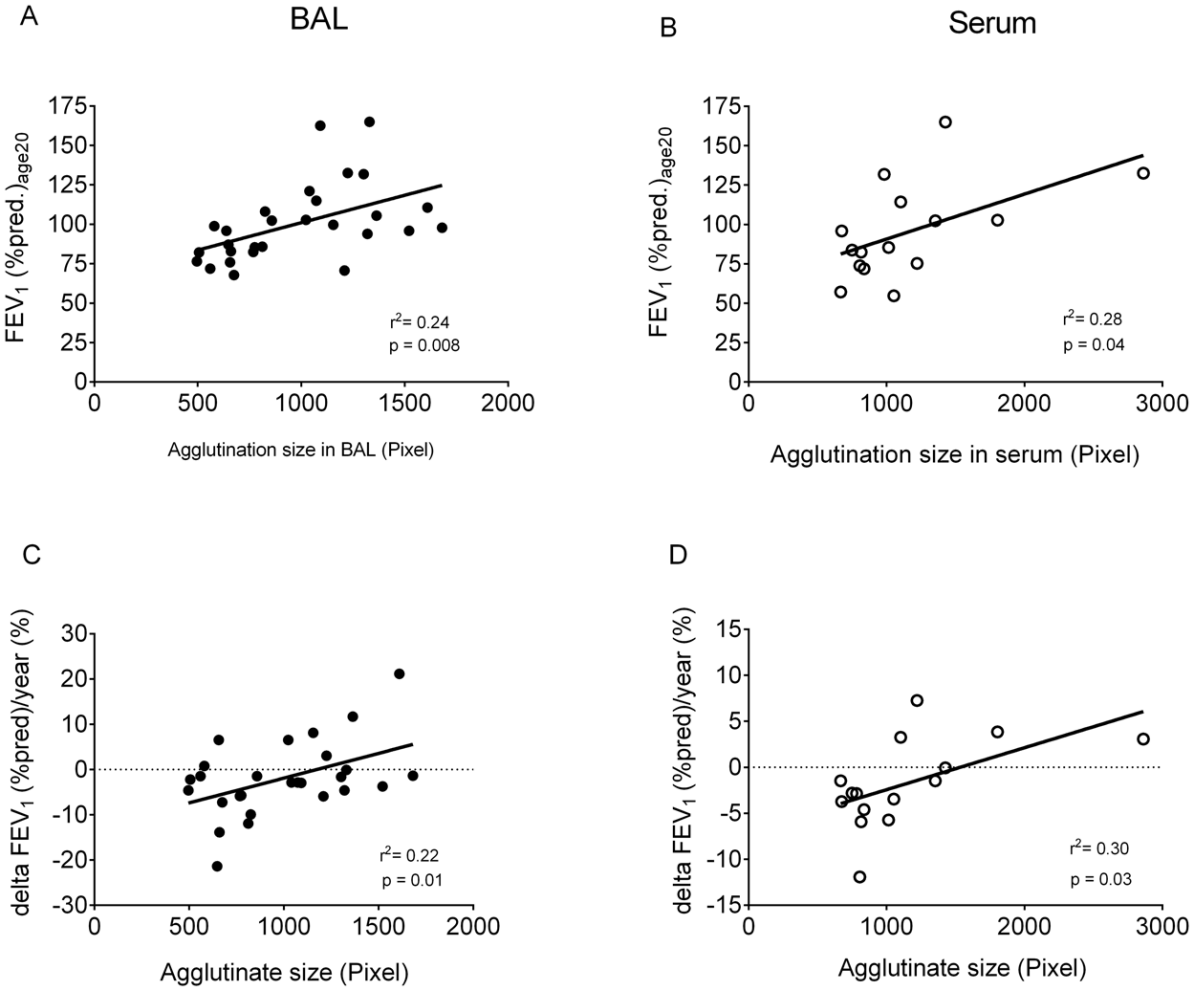
Correlation between agglutinate size and FEV1 (% pred.)_age20_ and ΔFEV1 (% pred.)/year. The graphs show on the x-axis the SP-A agglutination size in whole, non-size fractionated BAL (A, C) and serum (B, D) in Pixel and in the y-axis the FEV1 (% pred.)_age20_ (A, B), and accordingly ΔFEV1 (% pred.)/year (C, D). 28 BAL samples and 12 serum samples of CF patients were used for the graphs a and b and 26 BAL samples and 14 serum samples of CF patients could be included in the graphs C and D. The p-values analyzed by linear regression were 0.0076 (r^2^ = 0.2438) for BAL (A) and 0.0417 (r^2^ = 0.2819) for serum (B) as well as 0.0147 (r^2^ = 0.2156) for BAL (C) and 0.0343 (r^2^ = 0.3006) for serum (D).

To assess the relationship between organizational structure of SP-A, and clinical markers of disease severity, we assessed its link to FEV1; an important surrogate for the course of lung disease and survival. Mean FEV_1_ (% pred.)_age20_ was higher in patients with the “100” form, compared to those with more SP-A present as less complex forms (Fig. 2 c, d). This association was seen both in BAL and serum. In addition, the size of the agglutinates was positively correlated to lung function (see Fig. 3 a–d).

## Discussion

### Structural Diversity of SP-A Oligomeric Organization

The majority of SP-A in BAL of both healthy people and CF patients with mild lung disease is structurally organized as complex oligomeric forms. This result is similar to that we have obtained previously in healthy controls and patients with gastro-esophageal reflux disease [6]. The seven major oligomeric patterns of SP-A separated by gel chromatography were also not different between the patients groups studied, but were linked to disease severity in CF suggesting that more complex SP-A organization is linked to better clinical status.

For the first time we determined the SP-A oligomeric pattern in serum, in order to have a more convenient way of assessment than BAL. In serum multimeric forms were less frequently observed than in BAL (Table 2). Interestingly in serum, multimeric forms were present (above the lower level of 20% of total SP-) in about 70% of CF patients and 50% of bronchitis. As multimeric forms of SP-A are more active, we speculate that in CF and in part in bronchitis they may result from disease-induced systemic activation and enhanced formation. The lack of correlation between serum and lavage compartments may reflect no direct feed of the serum compartment from the alveolar space [22], [23].

### SP-A Oligomeric Structure and Function are Inter-connected in Humans and Superior Functional Activity is Linked to Better Course of Lung Function

The capacity of SP-A from serum and BAL to induce agglutination was linked to the organizational structure of naturally occurring macromolecular forms. The more complex the oligomeric forms were the better was SP-A dependent agglutination. The rank order was the same in BAL and serum. There are no *in vitro* data available which rank different oligomeric structures of SP-A with regard to interaction with micro-organisms, however results from studies with a mix of SPA1/SP-A2 and with SP-A2 which have a higher degree of oligomerization than SP-A1 demonstrate a better binding and aggregation of bacterial lipopolysaccharides by the more complex macromolecular forms of SP-A [4]. In studies utilizing SP-A derived from patients with alveolar proteinosis, which also has a macro-molecular organization as larger oligomers, the phagocytosis of *Staphylococcus aureus* by monocytes was enhanced by binding to C1qR [24]. Similarly, *Pseudomonas aeruginosa* and *A. fumigatus* agglutination, uptake and killing by phagocytic cells were superior [25]–[27].

### Factors that may Influence SP-A Oligomeric Composition

On the level of the protein, an important factor which may determine amount and structural organization of SP-A is the overall proteolytic activity present in alveolar lining fluid [6], [12]. In the present study we compared three patient groups, including mild CF patients with a normal lung function. In this group of CF patients neutrophils and elastase activity in BAL were elevated, although to a small extent. As expected on the basis of previous data [19], the functional activity of SP-A for agglutination, i.e. the size of agglutinates formed (Table 1) was reduced in CF patients. However the structural organization was not different to the comparison groups. This suggests that changes in the structural organization pattern of the SP-A may be much less sensitive to proteolytic activity than SP-A function. Thus the differences in functional activity of SP-A between the three groups in face of unchanged structural organization pattern may have been primarily the result of proteolysis of SP-A within multimers.

In CF patients, there was only a very weak direct correlation of elastase activity to the SP-A present as dimers/trimers (%), and none to higher oligomers. Clearly, the oligomeric pattern of SP-A present on itself was a considerably stronger determinant of its agglutination activity and was consistently linked to lung function. This observation is in agreement with results showing that the complex oligomerization of SP-A was essential for the collagen triple helix stability, protection against proteases, and SP-A-induced ligand aggregation [4]. Oligomeric pattern of SP-A may represent an additional factor of resilience to protect from loss of lung function.

The observed complexity of SP-Ás structural organization is likely based on the complex genetic organization of SP-A. Due to the presence of 2 SP-A genes, SFTPA1 and SFTPA2, in which more than 130 single nucleotide polymorphisms have been detected within the coding region, the resulting in a number of allelic variants is large [28], [29]. Additionally, many mRNA splice variants at variable stability and expression levels add to a significant number of peptides generated by one of the many haplotypes to be combined in the final SP-A multimer. For the analysis of associations between genetic SP-A variants and variation of SP-A protein concentrations and functional properties larger numbers of samples than investigated in this cohort are clearly necessary.

This study has some limitations. Although we investigated a rather large population with complex biochemical investigations, due to the significant number of different naturally occurring structural organizational forms of SP-A, the desirable number of subjects in some of the groups were lower than anticipated. Additional factors, like the atopy status, as suggested by Hickling et al. who found a higher percentage of complex oligomers like octadecamers in BAL from healthy persons compared to BAL from birch pollen from 11 allergic persons [7] may play an as yet undetermined role in influencing SP-A oligomeric composition. Lastly, more information on the generation and metabolism as well as in vivo function of the complex oligomeric forms may be helpful to explain the intriguing observations made in this study.

We selected to assess the multimeric state of SP-A by using Latex beads instead of bacteria, as the results can be better generalized and are more reproducible be obtained for several reasons; (1) not all bacterial strains behave in the same way, even within a species (e.g. [26], [30]) wide variation in agglutination is observed; (2) agglutination may be depended on the growth phase of the bacterial strain (own unpublished observation), and (3) the assay used has been investigated extensively previously in a previous study [19], allowing others to reproduce the findings independently. In addition, the purpose of the assay is to demonstrate the multi-valence of SP-A and compare it between subjects and not so much to assess different micro-organisms. Lastly, if bacteria would have been used, additional factors present in BAL or serum and interacting with other receptors on the bacterial surface might have interfered with such an assay.

In summary, our results demonstrate the presence of a wide range of naturally existing forms of SP-A oligomers, which are of functional relevance in patients with chronic lung diseases like cystic fibrosis. Such a close link between SP-A structure, its agglutination activity and patients lung function, underline the role of SP-A for human lung health.
